# Adaptive multi-view multi-label learning for identifying disease-associated candidate miRNAs

**DOI:** 10.1371/journal.pcbi.1006931

**Published:** 2019-04-01

**Authors:** Cheng Liang, Shengpeng Yu, Jiawei Luo

**Affiliations:** 1 School of Information Science and Engineering, Shandong Normal University, Jinan, China; 2 College of Computer Science and Electronic Engineering, Hunan University, Changsha, China; Ottawa University, CANADA

## Abstract

Increasing evidence has indicated that microRNAs(miRNAs) play vital roles in various pathological processes and thus are closely related with many complex human diseases. The identification of potential disease-related miRNAs offers new opportunities to understand disease etiology and pathogenesis. Although there have been numerous computational methods proposed to predict reliable miRNA-disease associations, they suffer from various limitations that affect the prediction accuracy and their applicability. In this study, we develop a novel method to discover disease-related candidate miRNAs based on Adaptive Multi-View Multi-Label learning(AMVML). Specifically, considering the inherent noise existed in the current dataset, we propose to learn a new affinity graph adaptively for both diseases and miRNAs from multiple similarity profiles. We then simultaneously update the miRNA-disease association predicted from both spaces based on multi-label learning. In particular, we prove the convergence of AMVML theoretically and the corresponding analysis indicates that it has a fast convergence rate. To comprehensively illustrate the prediction performance of our method, we compared AMVML with four state-of-the-art methods under different validation frameworks. As a result, our method achieved comparable performance under various evaluation metrics, which suggests that our method is capable of discovering greater number of true miRNA-disease associations. The case study conducted on thyroid neoplasms further identified a potential diagnostic biomarker. Together, the experimental results confirms the utility of our method and we anticipate that our method could serve as a reliable and efficient tool for uncovering novel disease-related miRNAs.

## Author summary

MiRNAs are a class of small non-coding RNAs that are associated with a variety of complex biological processes. Increasing studies have shown that miRNAs have close relationships with many human diseases. The prediction of the associations between miRNAs and diseases has thus become a hot topic. Although traditional experimental methods are reliable, they could only identify a limited number of associations as they are in general time-consuming and expensive. Consequently, great efforts have been made to effectively predict reliable disease-related miRNAs based on computational methods. In this study, we develop a novel method to discover potential miRNA-disease associations based on Adaptive Multi-View Multi-Label learning. Considering the inherent noise existed in the current dataset, we propose to learn a new affinity graph adaptively for both diseases and miRNAs from multiple biological data source, including miRNA sequence similarity, miRNA functional similarity and Gaussian interaction profile kernel similarity. Notably, our method is applicable to diseases without any known associated miRNAs and also obtains satisfactory results. The case study conducted on thyroid neoplasms further confirms the prediction reliability of the proposed method. Overall, results show that our method can predict the potential associations between miRNAs and diseases effectively.

## Introduction

MiRNAs are a group of short non-coding RNAs that mediate post-transcriptional gene silencing[1]. Accumulating evidence has proved that miRNAs play crucial roles in a variety of cancer-related pathways. Therefore, the identification of miRNA-disease associations can shed new light on understanding possible pathogenesis of diseases.

To compensate for the limitations of experiment-based approaches, a great number of computational models have been proposed to identify potential disease-related miRNAs in recent years[2]. Under the assumption that functionally similar miRNAs tend to be associated with phenotypically similar diseases, Jiang *et al*. prioritized the entire microRNAome for over a thousand diseases by constructing an integrated phenome-microRNAome network[3]. Chen *et al*. measured the global network similarity and inferred potential miRNA-disease interactions based on random walk with restart[4]. Shi *et al*. adopted a similar idea and further integrated the protein-protein interactions into the prediction process[5]. Chen *et al*. proposed a novel heterogeneous graph inference method by iteratively updating the association probability[6, 7]. Liu *et al*. constructed a heterogeneous network in which they integrated the miRNA-target gene and miRNA-lncRNA associations[8]. Specifically, the methods introduced above mainly predicted disease-related miRNAs by applying random walk algorithms to the reconstructed similarity networks[9]. Another family of prediction methods was generally based on network topological characteristics and also achieved remarkable performance. For instance, Zou *et al*. computed the similarity score based on walks of different lengths between the miRNA and disease nodes[10]. Sun *et al*. exploited the potential disease-related miRNAs based on known miRNA-disease network topological similarity[11]. You *et al*. proposed to measure the association score for a miRNA-disease pair by calculating the accumulative contributions from all paths between them[12]. Li *et al*. used DeepWalk to enhance the existing associations through a topology-based similarity measure[13]. Chen *et al*. computed the association possibility between a disease node and a miRNA node in the corresponding graphlet interaction isomers[14]. Although effective, these methods are sensitive to the change of the network topological structures, which might affect the prediction accuracy. Alternatively, prediction methods that were based on semi-supervised learning as well as supervised learning have been well developed. Xiao *et al*. introduced a graph regularized non-negative matrix factorization to effectively discover sparse miRNA-disease associations[15]. Both Chen *et al*. and Yu *et al*. adopted matrix completion to recover the potential missing miRNA-disease associations[16, 17]. Zeng *et al*. used a derivative algorithm structural perturbation method to estimate the link predictability with structural consistency as the indicator[18]. Chen *et al*. used an ensemble model where a sequence of weak learners were trained to collectively obtain a predicted association score[19]. Recently, we reconstructed the miRNA and disease similarity matrices based on global linear neighborhoods and then applied label propagation to predict potential associations between diseases and miRNAs[20, 21]. Chen *et al*. extracted novel feature vectors for both miRNAs and diseases to train a random forest classifier for the prediction task[22].

Although great efforts have been made to efficiently uncover potential miRNA-disease associations, most existing computational approaches still suffer from several limitations. Specifically, the inherent noise in the current datasets resulted in incomplete and sparse similarity matrices and thus inevitably affected the prediction accuracies of these methods. Moreover, the integration of multiple biological data sources in calculating the similarity matrices for both miRNAs and diseases was generally performed by averaging the input similarity information, which might lead to suboptimal results. Lastly, the predicted association scores from miRNA space and disease space were often updated separately during the learning process. To solve these problems, in this paper, we propose a novel Adaptive Multi-View Multi-Label(AMVML) learning framework to infer disease-related miRNAs. In particular, our method adaptively learns a new affinity graph for miRNAs and diseases respectively from multiple data sources (i.e. miRNA sequence similarity, Gaussian interaction profile kernel similarity and so on). In addition, we unify the optimization process for both disease space and miRNA space based on multi-label learning. The experimental results under several different evaluation metrics clearly demonstrate the superior performance of our method over previous methods. We further carry out a case study on thyroid cancer to identify potential prognostic biomarkers.

## Materials and methods

### Human miRNA-disease associations

**Fig 1:**
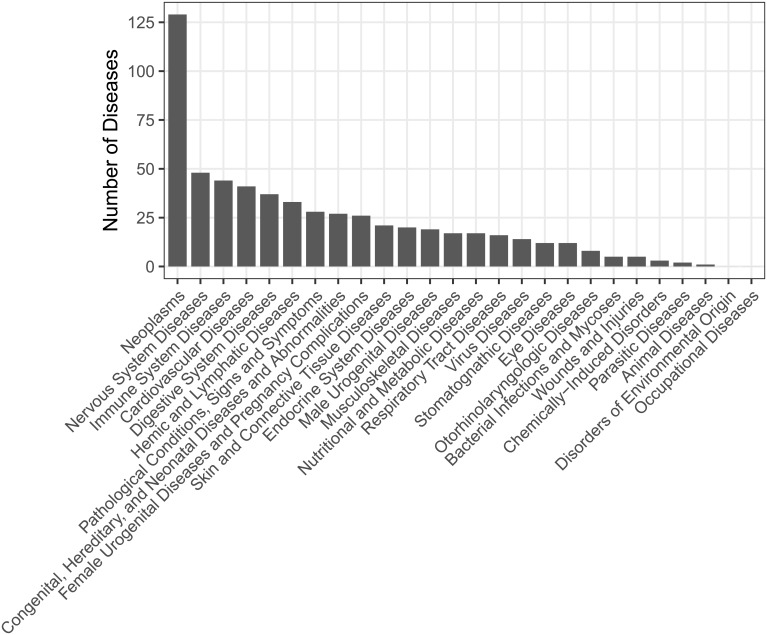
The type and distribution of the 328 diseases recorded in HMDD v2.0.

The known human miRNA-disease associations were retrieved from HMDD v2.0 database[23]. HMDD is a database for experimentally supported human miRNA and disease associations that were manually collected from all the miRNA-related publications in PubMed. Each entry in HMDD contains four items, i.e. miRNA name, disease name, experimental evidence for the miRNA-disease association and the publication PubMed ID. To keep consistent of data from different sources, we also downloaded the annotation information of 4796 human miRNAs released on March 2018 from miRBase[24]. We then downloaded the latest MeSH descriptors from the National Library of Medicine(https://www.nlm.nih.gov/) and only retained items from Category C for diseases, which resulted in 11572 unique items. After mapping the miRNA names and disease names involved in each association with miRBase records and MeSH descriptors, we finally obtained 6088 associations between 328 diseases and 550 miRNAs for subsequent analysis(S1 File). Specifically, we classified the 328 diseases based on the Diseases Categories provided in MeSH. For diseases belonging to multiple categories, we increased the count by one for each category accordingly. As a result (Fig 1, S2 File), we can see that most diseases recorded in HMDD were cancers. For convenience, we used a binary matrix ***Y*** ∈ ℝ^328×550^ to represent the miRNA-disease associations. For a given disease *i* and miRNA *j*, *Y*_*ij*_ *=* 1 if *i* is related to *j*, and *Y*_*ij*_ *=* 0 otherwise.

### Disease semantic similarity

As described in [25], the disease semantic similarity can be calculated based on Directed Acyclic Graphs (*DAGs*). Specifically, for a given disease *d*, its *DAG* is composed of three items, i.e. *DAG =* (*d*, *T*(*d*), *E*(*d*)), where *T*(*d*) represents *d* itself together with all its ancestor nodes, and *E*(*d*) contains all direct links connecting the parent nodes to child nodes. The contribution *D*_*d*_(*t*) of a disease *t* in a *DAG*_*d*_ to the semantics of disease *d* was defined as follows:

$$\left\{ \begin{array}{l} D_{d}\left(D\right)=1\\ D_{d}\left(t\right)=\text{max}\{0.5*D_{d}\left(t^{\prime }\right)|t^{\prime }\in children\;of\;t\}\;if\;t\neq d \end{array}\right.$$ (1)

The semantic similarity score between two diseases *i* and *j* can then be calculated by:

$$S\left(i,j\right)=\frac{\sum_{t\in T\left(i\right)\cap T\left(j\right)}\left(D_{i}\left(t\right)+D_{j}\left(t\right)\right)}{\sum_{t\in T\left(i\right)}D_{i}\left(t\right)+\sum_{t\in T\left(j\right)}D_{j}\left(t\right)}$$ (2)

Moreover, the similarity between a given disease *d* and a group of diseases *D*_*t*_ *=* {*d*_*t1*_, *d*_*t2*_,…, *d*_*tk*_} was defined by:

$$S\left(d,D_{t}\right)={\text{max}}_{1\leq i\leq k}\left(S\left(d,d_{ti}\right)\right)$$ (3)

Finally, we obtained the semantic similarities for each disease pair according to (Eq 2). We denoted the semantic similarity matrix as ***AD***^(1)^∈ ℝ^328×328^ where $AD_{ij}^{\left(1\right)}$ represents the semantic similarity between disease *i* and disease *j*(S3 File).

### MiRNA similarity measures

In this subsection, to comprehensively characterize similarities between miRNAs, we adopt three measures using different biological data sources for subsequent predictions[26].

#### MiRNA sequence similarity

The sequence information of all miRNAs were downloaded from miRBase[24]. We then used the "pairwiseAlignment" function in R package Biostrings to calculate a similarity score for each miRNA pair based on their entire mature sequences with a gap opening penalty of 5 and a gap extension penalty of 2. Moreover, to generate a substitution matrix for sequence alignment, we set the match score to 1 and the mismatch score to -1. Finally, the sequence similarity score obtained for each miRNA pair was further normalized to the range [0, 1] by the following equation:

$$Score\left(i,j\right)=\frac{Score\left(i,j\right)-Score_{\text{min}}}{Score_{\text{max}}-Score_{\text{min}}}$$ (4)

where *Score*_min_ and *Score*_max_ represent the minimum and maximum similarity score of all miRNA pairs. For simplicity, we use ***AM***^(1)^∈ ℝ^328×550^ to denote the sequence similarity matrix where $AM_{ij}^{\left(1\right)}$ represents the sequence similarity between miRNA *i* and miRNA *j*(S4 File).

#### MiRNA functional similarity

To measure the functional similarity for each miRNA pair, we followed the same pipeline presented in [25]. Let *DT*_*i*_ and *DT*_*j*_ denote the disease sets related to miRNA *i* and *j*, respectively, the functional similarity is calculated as follows:

$$MFS\left(i,j\right)=\frac{\sum_{1\leq p\leq \left|DT_{i}\right|}S\left(dt_{ip,}DT_{j}\right)+\sum_{1\leq q\leq \left|DT_{j}\right|}S\left(dt_{jq,}DT_{i}\right)}{\left|DT_{i}\right|+\left|DT_{j}\right|}$$ (5)

where *S*(*dt*, *DT*) is the same as that defined in (Eq 3). We use ***AM***^(2)^ ∈ ℝ^550×550^ to denote the miRNA functional similarity matrix and $AM_{ij}^{\left(2\right)}$ represents the functional similarity between miRNA *i* and miRNA *j*(S5 File).

#### MiRNA semantic similarity

As stated in previous section, the miRNA functional similarities could be obtained based on the overlap of miRNA-related diseases[15]. However, it is not applicable to miRNAs without any known associated diseases. Therefore, we here propose to use miRNA target information and the Gene Ontology (GO) annotations to better describe the miRNA semantic similarities. To this end, we first downloaded the experimentally-verified miRNA-gene interactions from mirTarBase[27], which contains 380639 interactions between 2599 miRNAs and 15064 genes. For each miRNA pair in our analysis, we maintained their target gene lists and then calculated the semantic similarity between the two corresponding gene groups by using the "clusterSim" function in the R package GOSemSim[28]. Specifically, the GO annotations were retrieved from the Bioconductor package "org.Hs.eg.db" and "BMA" method was used for combining semantic similarity scores of multiple GO terms[29]. Similarly, we used ***AM***^(3)^∈ ℝ^550×550^ to denote the miRNA semantic similarity matrix where $AM_{ij}^{\left(3\right)}$ represents the semantic similarity between miRNA *i* and miRNA *j*(S6 File).

### Gaussian interaction profile kernel similarity

Gaussian interaction profile kernel similarity has been widely used in previous studies and proved effective in measuring both miRNA and disease similarities. For a given miRNA *i* or disease *j*, its interaction profile *IP*(*m*_*i*_) or *IP*(*d*_*j*_) was a binary vector extracted from the *i*-th row or the *j*-th column of the association matrix *Y*. The kernel similarity between two miRNAs or two diseases could then be computed by:

$$KM\left(m_{i},m_{j}\right)=\text{exp}\left(-\beta_{m}{\left\|IP\left(m_{i}\right)-IP\left(m_{j}\right)\right\|}^{2}\right)$$ (6)

$$KD\left(d_{i},d_{j}\right)=\text{exp}\left(-\beta_{d}{\left\|IP\left(d_{i}\right)-IP\left(d_{j}\right)\right\|}^{2}\right)$$ (7)

where *β*_*m*_ and *β*_*d*_ are defined as follows:

$$\beta_{m}=\beta_{m}{}^{\prime }/\left(\frac{1}{550}\sum_{i=1}^{550}{\left\|IP\left(m_{i}\right)\right\|}^{2}\right)$$ (8)

$$\beta_{d}=\beta_{d}{}^{\prime }/\left(\frac{1}{328}\sum_{i=1}^{328}{\left\|IP\left(d_{i}\right)\right\|}^{2}\right)$$ (9)

where *β'*_*m*_ and *β'*_*d*_ are two parameters controlling the kernel bandwidth. As a result, we used ***AM***^(4)^∈ ℝ^550×550^ and ***AD***^(2)^∈ ℝ^328×328^ to represent the obtained Gaussian interaction profile similarity matrices for miRNAs and diseases, respectively.

### Adaptive multi-view multi-label learning for miRNA-disease association prediction

We summarize the notations used throughout this paper. Given a matrix *M*, *M*_*ij*_ and *M*_*i*_ represent its *ij*-th element and *i*-th row, respectively. The transpose of *M* is denoted by *M*^T^. *Tr*(*M*) denotes the trace of *M* and the Frobenius norm of *M* is represented as ||*M*||_*F*_. For a similarity matrix *S*, its Laplacian matrix *L*_*S*_ is defined as $L_{S}=D_{S}-\frac{S^{T}+S}{2}$, where *D*_*S*_ is a diagonal matrix with its *i*-th diagonal element equal to **∑**_*j*_(*S*_*ij*_ + *S*_*ji*_)/2.

#### Graph-based multi-label learning

Multi-label learning refers to the problems where an instance can be assigned to more than one category[30]. The graph-based multi-label learning framework is characterized by simultaneously exploiting the inherent correlations among multiple labels and the label consistency over the graph[31]. As a matter of fact, since each miRNA or disease could be associated with multiple diseases or miRNAs, this learning framework can be directly applied to solve the miRNA-disease association prediction problem by defining its objective function as follows:

$$\underset{F}{\text{min}}Tr\left({\left(F-Y\right)}^{T}\left(F-Y\right)\right)+\mu Tr\left(F^{T}LF\right)+\nu Tr\left(FCF^{T}\right)$$ (10)

where *L* and *C* are the normalized Laplacian matrices corresponding to the similarity matrices of miRNAs and diseases, respectively. *μ* and *v* are two non-negative trade-off parameters. By differentiating the objective function with respect to *F*, (Eq 10) can be efficiently solved by a Sylvester equation.

#### AMVML

**Fig 2:**
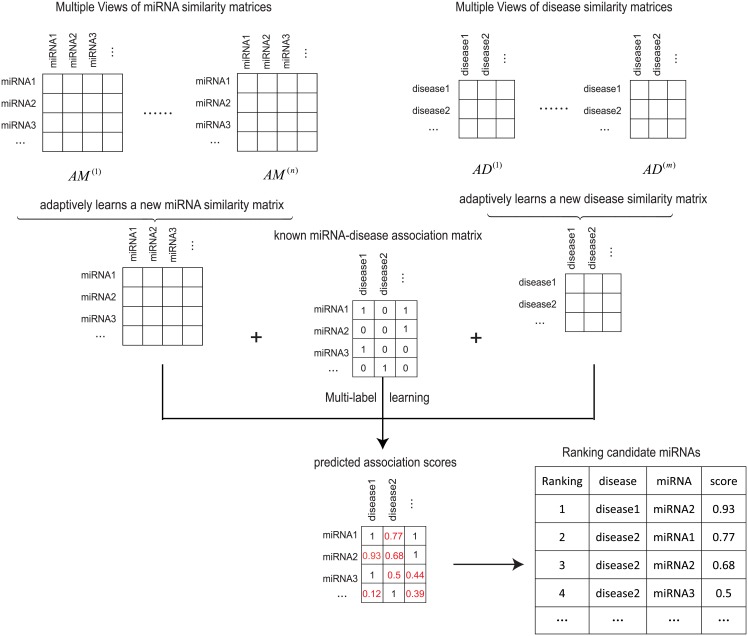
The overall workflow of AMVML. Our method first learns two new affinity graphs for miRNAs and diseases from multiple sources of biological datasets, respectively. It then updates the miRNA-disease association information simultaneously based on multi-label learning framework. It repeats this process until the algorithm converges and finally outputs the prediction results.

The graph-based multi-label learning provides us a unified framework to collaboratively update the prediction results from miRNA space and disease space, which successfully solves the last issue mentioned above. However, we still suffer from the inherent noises in the existing datasets as well as the lack of appropriate methods to integrate datasets from multiple biological data sources. To conquer these limitations, we propose a new objective function which can adaptively learns new affinity graphs for miRNAs and diseases given similarity information obtained from multiple views, respectively(Fig 2). Moreover, instead of explicitly assigning weights for each view, our method can also perform self-conducted weight learning during the optimization process[32]. Specifically, let *n* be the number of views in miRNA space and *AM*^(1)^, *AM*^(2)^,…, *AM*^(*n*)^ be the corresponding miRNA similarity matrix of each view, where ***AM***^(*u*)^ ∈ ℝ^*p*×*p*^ (1≤*u*≤*n*). Similarly, let *m* be the number of views in disease space and *AD*^(1)^, *AD*^(2)^,…, *AD*^(*m*)^ be the corresponding disease similarity matrix of each view, where ***AD***^(*v*)^ ∈ ℝ^*q*×*q*^ (1≤*v*≤*m*). *p* and *q* are the number of miRNAs and diseases, respectively. Our objective is to obtain the predicted association matrix *F* as well as two optimal similarity matrices *SD* and *SM* by considering multiple input views in both disease and miRNA spaces simultaneously, which is formulated as follows:

$$\begin{array}{l} \underset{SD,SM,F}{\text{min}}\sum_{v=1}^{m}w_{D}^{\left(v\right)}{\left\|SD-AD^{\left(v\right)}\right\|}_{F}^{2}+2\alpha \left(F^{T}L_{SD}F\right)+\\ \;\sum_{u=1}^{n}w_{M}^{\left(u\right)}{\left\|SM-AM^{\left(u\right)}\right\|}_{F}^{2}+2\beta \left(FL_{SM}F^{T}\right)+{\left\|F-Y\right\|}_{F}^{2}\\ s.t.\;SD_{i}1=1,0\leq SD_{ij}\leq 1,SM_{i}1=1,0\leq SM_{ij}\leq 1,F\in {\mathbb{R}}^{q\times p} \end{array}$$ (11)

where *L*_*SD*_ and *L*_*SM*_ are the corresponding Laplacian matrices for *SD* and *SM*, respectively. **1** is a column vector with all elements equal to 1.$w_{D}^{\left(v\right)}$ and $w_{M}^{\left(u\right)}$ are weight parameters for the *v*-th view of disease similarity and *u*-th view of miRNA similarity defined by:

$$w_{D}^{\left(v\right)}=1/\left(2{\left\|SD-AD^{\left(v\right)}\right\|}_{F}\right)$$ (12)

$$w_{M}^{\left(u\right)}=1/\left(2{\left\|SM-AM^{\left(u\right)}\right\|}_{F}\right)$$ (13)

As can be seen from Eqs (12) and (13), the two weight factors $w_{D}^{\left(v\right)}$ and $w_{M}^{\left(u\right)}$ are seamlessly coupled with ||*SD—AD*^(*v*)^||_*F*_ and ||*SM—AM*^(*u*)^||_*F*_, respectively. Intuitively, if the *v*-th or *u*-th view is good, then ||*SD—AD*^(*v*)^||_*F*_ or ||*SM—AM*^(*u*)^||_*F*_ should be small and thus the learnt $w_{D}^{\left(v\right)}$ or $w_{M}^{\left(u\right)}$ will be assigned a larger weight accordingly. As a result, $w_{D}^{\left(v\right)}$ and $w_{M}^{\left(u\right)}$ are updated adaptively in terms of the quality of the corresponding view during each iteration, which essentially makes the optimization of our objective function a self-weighted learning process. In the next part, we propose an efficient algorithm to solve (Eq 11).

#### Optimization

It is difficult to directly solve (Eq 11) as it involves three variables. Therefore, we iteratively optimize one variable by fixing the others[33].

1. Solving *SD*. When *SM* and *F* are fixed, (Eq 11) becomes:
  $$\sum_{v=1}^{m}w_{D}^{\left(v\right)}{\left\|SD-AD^{\left(v\right)}\right\|}_{F}^{2}+2\alpha \left(F^{T}L_{SD}F\right),s.t.\;SD_{i}1=1,0\leq SD_{ij}\leq 1$$ (14)
  (Eq 14) can be further transformed into:
  $$\underset{0<SD_{ij}<1,SD_{i}1=1}{\text{min}}\sum_{v=1}^{m}w_{D}^{\left(v\right)}\sum_{i,j=1}^{q}{\left(SD_{ij}-AD_{ij}^{\left(v\right)}\right)}^{2}+\alpha \sum_{i,j=1}^{q}{\left\|f_{i}-f_{j}\right\|}_{2}^{2}SD_{ij}$$ (15)
  Since (Eq 15) is independent for different *i*, we can optimize each row separately:
  $$\underset{0<SD_{ij}<1,SD_{i}1=1}{\text{min}}\sum_{j=1}^{q}\sum_{v=1}^{m}w_{D}^{\left(v\right)}{\left(SD_{ij}-AD_{ij}^{\left(v\right)}\right)}^{2}+\alpha \sum_{j=1}^{q}{\left\|f_{i}-f_{j}\right\|}_{2}^{2}SD_{ij}$$ (16)
  Denoting *z*_*i*_ as a vector with its *j*-th element $z_{ij}={\left\|f_{i}-f_{j}\right\|}_{2}^{2}$ and similarly for *SD*_*i*_ and $AD_{i}^{\left(v\right)}$, (Eq 16) could be rewritten as:
  $$\underset{SD_{i}>0_{q}^{T},SD_{i}1=1}{\text{min}}{\left\|SD_{i}-\left(\sum_{v=1}^{m}w_{D}^{\left(v\right)}AD_{i}^{\left(v\right)}-\frac{\alpha }{2}z_{i}\right)/\sum_{v=1}^{m}w_{D}^{\left(v\right)}\right\|}_{2}^{2}$$ (17)
  Problem (17) can be solved efficiently by an iterative algorithm[34].
2. Solving *SM*. Similarly, when *SD* and *F* are fixed, (Eq 11) becomes:
  $$\sum_{u=1}^{n}w_{M}^{\left(u\right)}{\left\|SM-AM^{\left(u\right)}\right\|}_{F}^{2}+2\beta \left(FL_{SM}F^{T}\right),s.t.\;SM_{i}1=1,0\leq SM_{ij}\leq 1$$ (18)
  By following the same optimization process for *SD*, we can derive the solution for problem (18) as follows:
  $$\underset{SM_{i}>0_{p}^{T},SM_{i}1=1}{\text{min}}{\left\|SM_{i}-\left(\sum_{u=1}^{n}w_{M}^{\left(u\right)}AM_{i}^{\left(u\right)}-\frac{\beta }{2}z_{i}\right)/\sum_{u=1}^{n}w_{M}^{\left(u\right)}\right\|}_{2}^{2}$$ (19)
3. Solving *F*. When *SD* and *SM* are fixed, (Eq 11) degenerates to:
  $$\underset{F}{\text{min}}2\alpha \left(F^{T}L_{SD}F\right)+2\beta \left(FL_{SM}F^{T}\right)+{\left\|F-Y\right\|}_{F}^{2}$$ (20)
  Taking the derivative of (Eq 20) with respect to *F* and setting it to zero, we could obtain:
  $$\left(\beta L_{SM}+I\right)F+\alpha FL_{SD}=Y$$ (21)
  (Eq 21) is a Sylvester equation and could be solved directly. We summarized the overall procedure in **Algorithm 1**. Besides, the datasets and source code of AMVML are freely available at https://github.com/alcs417/AMVML.

**Algorithm 1:** AMVML

**Input:** miRNA similarity matrices of *n* views {*AM*^(1)^, *AM*^(2)^,…, *AM*^(*n*)^}, disease similarity matrices of *m* views {*AD*^(1)^, *AD*^(2)^,…, *AD*^(*m*)^}, known association matrix *Y* ∈ ℝ^*q*×*p*^, parameter *α* and *β*.

**Output:** Predicted association matrix *F*.

1. Initialize the weights of each view for both miRNAs and diseases by $w_{D}^{\left(v\right)}=\frac{1}{m},w_{M}^{\left(u\right)}=\frac{1}{n}$.

2. Repeat:

3.  Repeat:

4.   Update *SD* by solving problem (Eq 17).

5.   Update *SM* by solving problem (Eq 19).

6.   Update *F* with (Eq 21).

7.  Until convergence

8. Update $w_{D}^{\left(v\right)},w_{M}^{\left(u\right)}$ according to Eqs (12) and (13)

9. Until convergence

10. Return *SD*, *SM* and *F*

#### Theoretical convergence analysis

We prove the convergence of **Algorithm 1** by the following theorem.

**Theorem 1**. The iterative optimization process in **Algorithm 1** can monotonically decrease the objective function value of (Eq 11) until convergence.

*Proof*. By fixing *SM* and *F*, the optimization for *SD* in (Eq 17) is a quadratic programming problem[35]. Specifically, the Hessian matrix of the Lagrange function of (Eq 17) is positive definite, i.e. **2*I*** ∈ ℝ^*q*×*q*^. Therefore, we arrive at:

$$\Omega \left(SM,SD^{t},F\right)>\Omega \left(SM,SD^{t+1},F\right)$$ (22)

Similarly, we could prove that the optimization for *SM* by fixing the other variables is also convex and we could obtain that:

$$\Omega \left(SM^{t},SD,F\right)>\Omega \left(SM^{t+1},SD,F\right)$$ (23)

By fixing *SD* and *SM*, the optimization function for updating *F* is also convex[36]. Moreover, since *βL*_*SM*_*+I* is positive definite and *βL*_*SD*_ is positive semi-definite, the eigenvalues *σ*_1_, *σ*_2_,…,*σ*_*l*_ of *βL*_*SM*_*+I* and *ξ*_1_, *ξ*_2_,…,*ξ*_*k*_ of *βL*_*SD*_ satisfy the inequality *σ*_*i*_*+ξ*_*j*_> 0 (*i =* 1,…,*l*; *j =* 1,…,*k*), which guarantees that there is a unique solution to (Eq 21). As a result, we could obtain:

$$\Omega \left(SM,SD,F^{t}\right)\geq \Omega \left(SM,SD,F^{t+1}\right)$$ (24)

As demonstrated in the above analysis, **Algorithm 1** can monotonically decrease the objective function value of (Eq 11) in each iteration until it converges.

## Results

### Performance evaluation

**Fig 3:**
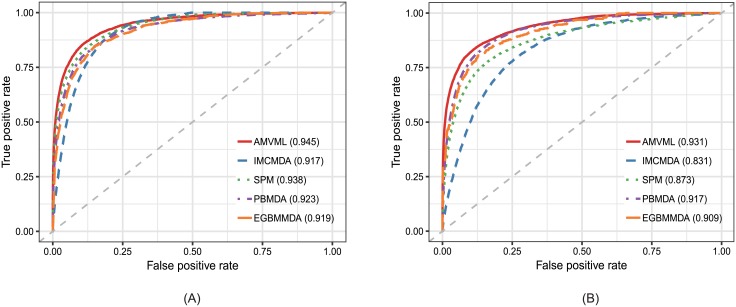
The comparison results between our method and the other four methods in terms of (A) global LOOCV; (B) five-fold cross-validation.

To systematically evaluate the performance of our method and illustrate its superiority over existing alternatives, we compared AMVML with fourstate-of-the-art methods, i.e. IMCMDA[37], SPMMDA[38], PBMDA[12] and EGBMMDA[19] under several evaluation metrics. All these methods have been proved effective in predicting reliable disease-associated miRNAs. First of all, we adopted the global Leave-One-Out Cross-Validation(LOOCV) and five-fold cross-validation to test the general prediction performance. Specifically, in the framework of global LOOCV, each known miRNA-disease association was selected as a test sample while the remaining associations were considered as training samples. For five-fold cross-validation, all known miRNA-disease associations were randomly divided into five subsets and each subset was chosen as the test samples. Besides, the five-fold cross-validation was repeated 10 times to eliminate the potential bias caused by the sample division. The prediction performance was illustrated by Receiver Operating Characteristic(ROC) curve and the accuracy was quantified by the Area Under the ROC Curve(AUC). As shown in Fig 3, AMVML achieved the highest accuracy among all methods in both global LOOCV and five-fold cross-validation.

**Fig 4:**
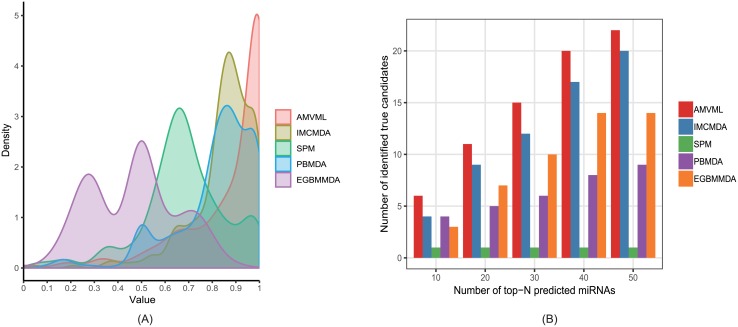
(A) The comparison results between our method and the other four methods in terms of LODOCV. As shown in the density plot, AMVML lies in the rightmost position of the figure, indicating a superior performance over the other methods; (B) The number of identified true positives in the latest HMDD by all methods.

Next, we employed another evaluation metric called Leave-One-Disease-Out Cross-Validation(LODOCV) to verify the prediction performance when no prior information is available. Specifically, for each disease *d*, we removed all known miRNAs associated with *d* and carried out predictions based on miRNA association information of the other diseases. Since there are no known associations for each tested disease in advance, LODOCV is more difficult than global LOOCV and five-fold cross-validation. We calculated an AUC value for each disease in LODOCV and thus obtained a vector consisting of 328 AUC values for each method. We then demonstrated the comparison results by density plots (Fig 4A). As a result, the AUC values obtained by our method mainly concentrated over the interval [0.9, 1], indicating a better performance than that of the other methods in terms of LODOCV. Wilcoxon signed-rank test further confirmed the statistical significance of the comparison results (Table 1).

**Table 1:** Statistical significance of differences in performance between AMVML and the other four methods in terms of LODOCV. *P-values* were calculated by Wilcoxon signed rank test.

	IMCMDA	SPM	PBMDA	EGBMMDA
*p-value*	2.67e-10	1.36e-41	2.86e-13	2.18e-21

Lastly, we conducted experiments on real datasets to further demonstrate the prediction ability of our method. To this end, we first downloaded the older version of HMDD (v1.0) which contains 1474 known associations between 129 diseases and 280 miRNAs after filtering (S7 File). Compared to HMDD v1.0, there were 4614 (i.e. 6088–1474) new miRNA-disease associations, 199 (i.e. 328–129) new diseases and 270 (i.e. 550–280) new miRNAs involved in HMDD v2.0. In particular, among the 4614 newly recorded associations in HMDD v2.0, 2445 associations were related with miRNAs and diseases already existed in HMDD v1.0, while 2169 associations were related with either new miRNAs or new diseases only contained in HMDD v2.0. Moreover, the degree distribution of miRNAs as well as that of diseases for the 4614 associations indicating that only a minority of these associations were related with highly connected miRNAs and diseases (S1 Fig). We then applied each method on HMDD v1.0 and validated the prediction results by the 4614 associations newly added in HMDD v2.0. Therefore, for each method, the greater the number of true positives predicted, the better the performance. Specifically, we compared the number of true positives in the top-*N* miRNAs predicted by each method with *N* ranging from 10 to 50 and an interval of 10. As exhibited in Fig 4B, AMVML obtained greater number of validated disease-associated miRNAs than the other methods. Similar results were also obtained with increased *N* and larger intervals (S2 Fig). Taken together, the experimental results under various evaluation metrics proved the effectiveness of our method.

### Parameter analysis

**Fig 5:**
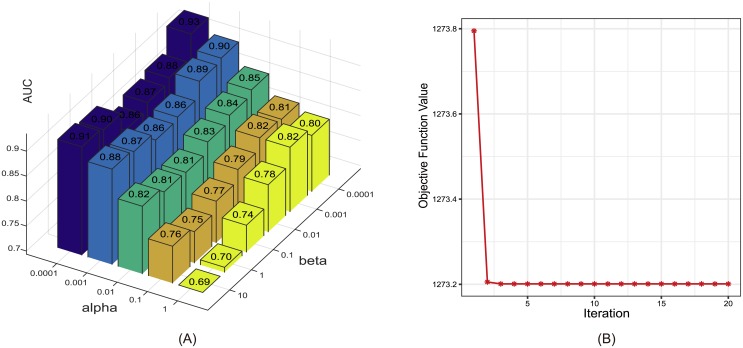
(A) The influence of the two parameters alpha and beta on the prediction accuracy of five-foldcross-validation; (B) The convergence rate of AMVML.

There were two trade-off parameters *α* and *β* in our method which balance the learned similarity matrices and the predicted association matrix. Generally, since our objective function is a minimization problem, setting a large value to *α* or *β* indicates a large impact of the label consistency between diseases or miRNAs on the learned disease or miRNA similarity matrix. To show a reasonable searching range of these two parameters as well as a general trend of the prediction performance affected by varying their values, in this subsection, we analyzed their influences on the prediction accuracy in terms of five-fold cross-validation (Fig 5A). Similar trends were also observed in global LOOCV. In particular, when *β* was fixed, the smaller the *α*, the better the performance. In contrast, when *α* was fixed, the performance varied in a "U" shape with the change of *β*. We can see that the proposed method reached the best performance when both *α* and *β* were equal to 1e-4.

### Convergence speed in practice

As described in previous section, we have theoretically proved the convergence of our algorithm. Here we investigated the convergence rate of our method by analyzing the variations of the objective function value in (Eq 11) with respect to the number of iterations. As demonstrated in Fig 5B, the objective function value reached a steady state within 5 iterations, indicating a fast convergence rate of our method.

### Case study

**Fig 6:**
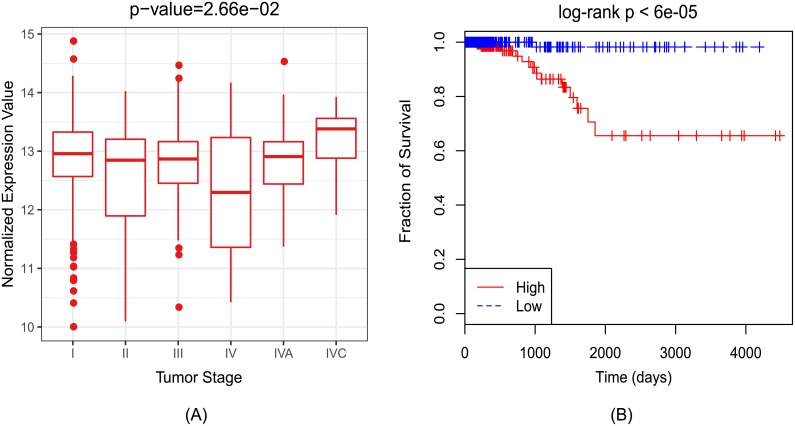
(A) The expression profiles of hsa-mir-181a-2 at different tumor stages. P-value was calculated by one-way ANOVA test; (B) Kaplan-Meier survival analysis using hsa-mir-181a-2 in thyroid cancer. The patients were divided into two groups ’High’ and ’Low’ according to their expression level of hsa-mir-181a-2 against the sample mean. As observed, patients with lower expression levels were at lower risk level.

In this section, we conducted a case study on thyroid neoplasms to identify potential miRNA biomarkers for this disease. The overall prediction results and the differential expression analysis for several other diseases were also provided on Github (https://github.com/alcs417/AMVML). Thyroid cancer is the most common endocrine cancer and its incidence rate has increased rapidly over recent years[39]. We first downloaded the miRNA expression profiles together with the clinical information of thyroid carcinoma from GDC data portal (https://portal.gdc.cancer.gov/projects/TCGA-THCA). Concretely, the downloaded data contained 506 tumors samples and 59 normal samples and each sample measured the expression level of 1881 miRNAs. We then applied our method on the given disease to obtain the top-10 predicted miRNAs(Table 2). Specifically, we evaluated the classification power of these miRNAs in differentiating tumor samples from normal samples according to their expression profile and the results of five-fold cross-validation illustrated that they could achieve a mean classification accuracy of 0.983(S3 Fig). Next, we calculated for each miRNA the fold-change as well as the statistical significance of differential expression using the R package edgeR (Table 2)[40]. Besides, we searched in another two databases dbDEMC and miR2Disease to see if the predicted miRNAs were also recorded in them[41, 42]. dbDEMC is an integrated database that designed to store and display differentially expressed miRNAs in human cancers detected by high-throughout methods while miR2Disease is a manually curated database providing information about miRNA deregulation in various human diseases. As a result, the expression level of the top predicted miRNA hsa-mir-181a-2 was significantly altered between tumor samples and normal samples (log2 fold-change > 1 and adjusted *p-value*< 0.05), which is consistent with the records in both db2DEMC and miR2Disease. Therefore, we further checked whether this miRNA could serve as a potential biomarker for thyroid cancer. Specifically, we carried out one-way ANOVA test to validate whether its expression level at different tumor stages also significantly altered. The tumor stages of all patients were retrieved from the clinical information and there were six pathologic stages after filtering. As expected, the expression level of hsa-mir-181a-2 varied significantly among different stages (Fig 6A). Furthermore, the Kaplan-Meier survival analysis confirmed that the survival rates of patients were also significantly related with its expression level (Fig 6B)[43]. Taken together, our results provided new evidence for the functional role of hsa-mir-181a-2 in the development of thyroid cancer.

**Table 2:** Top-10 miRNAs predicted to be associated with thyroid neoplasms by AMVML.

Ranking	miRNA	logFC	FDR	Evidence
1	hsa-mir-181a-2	1.47	1.55e-41	dbDEMC; miR2Disease
2	hsa-mir-205	2.01	2.76e-06	dbDEMC
3	hsa-mir-143	-0.26	1.51e-01	dbDEMC
4	hsa-mir-145	-0.4	1.52e-03	dbDEMC
5	hsa-mir-101-2	-0.044	1.00	unconfirmed
6	hsa-mir-34a	2.23	1.86e-59	dbDEMC;miR2Disease
7	hsa-mir-133a-1	-0.62	2.37e-03	dbDEMC
8	hsa-mir-99a	-0.42	2.24e-06	dbDEMC
9	hsa-mir-218-1	-0.61	2.43e-08	dbDEMC
10	hsa-mir-34c	-0.14	7.20e-01	unconfirmed

## Discussion

Identification of disease-associated miRNAs has drawn much attention during the past decade and it still remains a challenging task. In this study, we proposed a novel computational framework to effectively uncover the potential links between miRNAs and diseases. Our method integrated datasets from multiple sources and adaptively learned two new similarity graphs. Specifically, instead of assigning predetermined weight values to each input similarity matrix, the proposed method automatically updated the view weights according to the reliability of each view. It is also worth mentioning that our method could be easily extended if there are new data sources available. Besides, our method could simultaneously update the prediction results from both disease space and miRNA space. The convergence of our method has been proved both theoretically and experimentally. To demonstrate the utility of our method, we compared AMVML with five state-of-the-art methods and the experimental results confirmed the superiority of our method. We then applied our method on thyroid cancer and found that hsa-mir-181a-2 could be a potential prognostic biomarker. Notably, our method is not limited to discover miRNAs for which an association is already known between its paralogous miRNA and the same disease. In essence, as a semi-supervised learning model, our method could fully take advantage of the limited number of known miRNA-disease associations together with multiple sources of biological datasets to reliably predict novel associations. Therefore, we anticipate that our method could serve as an effective tool for miRNA-disease association prediction.

The superior performance of our model can be largely attributed to the following two reasons. First, the consensus similarity matrices obtained from multiple biological datasets based on multi-view learning for both miRNAs and diseases are more robust to outliers and noises compared to existing methods. Second, the graph-based multi-label learning unified the two prediction spaces into one optimization framework, which enhances the inherent correlations between miRNAs and diseases from the label-consistency point of view. Nevertheless, our method still has some limitations. Specifically, there are two parameters *α* and *β* in the objective function that need to be tuned in advance, and it is a non-trivial task to find the best combination of the two parameters. In addition, although our method can adaptively learn a new affinity graph from different data sources, the integration of unreliable similarity matrices might weaken the overall prediction accuracy.

## Supporting information

S1 Fig The degree distribution of (A): Diseases and (B): miRNAs. (EPS)

S2 Fig The number of identified true positives in HMDD v2.0 by all methods with N ranging from (A) 200 to 1000; and (B) 2000 to 10000. (EPS)

S3 Fig Five-fold cross-validation results obtained by taking the top 10 predicted miRNAs as features. (EPS)

S1 File The 6088 miRNA-disease associations obtained from HMDD v2.0. (XLSX)

S2 File The number of diseases in each disease typecontained in HMDD v2.0. (XLSX)

S3 File Disease semantic similarity matrix. (XLSX)

S4 File MiRNA sequence similarity matrix. (XLSX)

S5 File MiRNA functional similarity matrix. (XLSX)

S6 File MiRNA semantic similarity matrix. (XLSX)

S7 File The 1474 miRNA-disease associations obtained from HMDD v1.0. (XLSX)
